# Research on LSTM-based spatial target trajectory forecasting enhanced by attention mechanisms

**DOI:** 10.1371/journal.pone.0356376

**Published:** 2026-08-21

**Authors:** Qingshan Luo, Jiahao Ji, Tao Yang, Yurui Xu, Yunsheng Yao

**Affiliations:** 1 College of Information and Control Engineering, Institute of Disaster Prevention, Sanhe, Hebei, China; 2 Hebei Key Laboratory of Seismic Disaster Instrument and Monitoring Technology, Sanhe, Hebei, China; 3 National Space Science Center, Chinese Academy of Sciences, Beijing, China; 4 Xingtai University, Xingtai, China; University of Zaragoza, SPAIN

## Abstract

To address the strong dependence of space object orbit prediction on physical models and initial conditions, as well as the difficulty of completely eliminating prediction errors, this study proposes a satellite orbit prediction correction method that integrates an attention mechanism with a long short-term memory (LSTM) network. Taking the LAGEOS satellite as the research object, the proposed method uses position error, velocity, and acceleration features extracted from historical orbital data to train a deep learning model for predicting one-day-ahead orbital errors and correcting the SGP4 orbit prediction results. The experimental results show that the ATLSTM model outperforms the LSTM, support vector machine (SVM), back propagation neural network (BP), and bidirectional long short-term memory (BiLSTM) models in both orbital error prediction and correction. The residual ratios of ATLSTM in the X, Y, and Z axes are reduced to 3.68%, 4.77%, and 2.37%, respectively, effectively improving the accuracy of satellite orbital error prediction. Further analysis indicates that a reasonable setting of the number of neurons helps improve model performance, while the prediction difficulty increases with the extension of the prediction duration, suggesting that the ATLSTM model is more suitable for short-term orbital error prediction and correction. In addition, validation results for satellites at different orbital altitudes demonstrate that the proposed model has certain generalization capability. In summary, combining deep learning methods with physical orbital models can effectively improve the accuracy of space object orbit prediction and provides an effective approach for orbital error prediction, space situational awareness, and collision warning.

## 1 Introduction

With the rapid development of global space technology, the number and types of space objects have been continuously increasing [1], including not only operational satellites and space stations but also a large amount of orbital debris [2–4]. The accurate prediction of the orbits of these space objects plays a crucial role in space mission planning and collision avoidance. Therefore, achieving efficient and precise Space Situational Awareness (SSA) is of great importance [5]. Since the orbits of space objects are influenced by complex perturbative forces, such as the Earth’s gravitational field, third-body perturbations, atmospheric drag, and solar radiation pressure [6], these effects are particularly significant for objects in low Earth orbit (LEO) and medium Earth orbit (MEO). External environmental disturbances, including atmospheric density variations and solar activity cycles, combined with the simplifications in orbital dynamics modeling, can lead to considerable error accumulation during long-term propagation. As a result, traditional dynamics-based orbit prediction models often exhibit substantial inaccuracies.Therefore, enhancing the accuracy of orbit prediction has become a major focus and a critical research direction in the field of modern space situational awareness.

To effectively improve the accuracy of space object orbit prediction, researchers have long focused on advancing orbital dynamics modeling and prediction algorithms. Currently, orbit prediction methods can be broadly classified into two categories. The first category comprises traditional mechanics-based methods, such as analytical models (e.g., SGP4) and numerical integration, which calculate the evolution of orbital states by establishing comprehensive perturbation models. These methods offer strong physical interpretability and a solid theoretical foundation.For example, Srivastava et al. proposed a geometry-based solar interruption prediction model, which predicts satellite orbits by applying a propagation operator to the ground station antenna state vector [7]. Liang et al. developed an improved orbital aerodynamics analysis model that incorporates a small-lobe quasi-specular scattering mode, significantly enhancing the modeling accuracy of aerodynamic coefficients [8]. Elhag et al. applied an Unscented Kalman Filter (UKF) to integrate TLE data with propagated orbital states, dynamically correcting orbital errors and improving orbit accuracy and position prediction performance [9]. However, traditional orbital dynamics modeling approaches such as the Simplified General Perturbations 4 (SGP4) model based on Two-Line Element (TLE) data are limited by simplified physical assumptions and uncertainties in environmental parameters. TLE data do not inherently include information about orbital accuracy, which restricts their reliability for high-precision applications [10–13]. In addition, the SGP4 model shows deficiencies when dealing with complex perturbative effects, such as atmospheric drag and high-order terms in the Earth’s gravitational field, leading to considerable prediction errors for both low-Earth orbit (LEO) and high-Earth orbit (HEO) satellites [14,15].

Compared with traditional methods, deep learning techniques particularly neural networks and hybrid models have gained increasing attention due to their effectiveness in processing time-series data and capturing the nonlinear and complex dynamics of satellite orbits [16,17]. In particular, the Long Short-Term Memory (LSTM) network demonstrates strong advantages in modeling temporal dependencies, while the attention mechanism excels in identifying and extracting key features, together providing a new framework for orbital error modeling.For example, Zhu et al. proposed an LSTM-based orbit prediction approach that learns from historical orbital data, successfully reducing the 20-day prediction error from approximately 300 km to less than 5 km [18]. Yang bypassed traditional physics-based modeling by directly learning the orbital evolution patterns of satellites through an LSTM network, and further optimized the model using the Firefly Algorithm, improving the one-day prediction accuracy to the hundred-meter level [19]. Zhang et al. proposed an error-driven LSTM model, which reduced the three-axis prediction errors of the Ajisai satellite to 16.87%, 17.66%, and 19.58% of their original values, respectively [20,21]. Moreover, Peng et al. investigated various machine learning approaches—including artificial neural networks (ANN), support vector machines (SVM), Gaussian processes (GP), and Extended Kalman Filters (EKF)—all of which demonstrated improvements in orbit prediction accuracy to varying degrees [22–28].

Although the above studies have demonstrated the feasibility of applying machine learning to aerospace applications, their prediction methods mainly rely on historical data or correction strategies based on past errors, and the extraction of key features is not sufficiently deep. This often results in large deviations in prediction accuracy. To address this issue, this study proposes a hybrid prediction method that combines a LSTM neural network with an attention mechanism to improve the accuracy of satellite orbit prediction.

This paper takes the LAGEOS satellite as the research subject and proposes an orbit error prediction and correction method that integrates a traditional orbital dynamics model with a deep learning model. Using the historical orbital data from the previous seven days, the orbital positions obtained from the precise ephemeris are compared with those derived from the SGP4 model combined with TLE to calculate the satellite’s position errors. These errors, together with the satellite’s velocity and acceleration, are used as inputs to the deep learning model.An Attention-Enhanced Long Short-Term Memory (ATLSTM) model is constructed to predict the satellite’s position errors for the eighth day, which are then used to correct the orbit forecast and obtain the refined orbital prediction. To verify the effectiveness of the proposed model, the LSTM and SVM models are selected for comparison, and the prediction accuracy of the ATLSTM model is analyzed under different training durations and numbers of hidden units.

## 2 Data sources and error analysis

This paper takes the LAGEOS satellite as the research subject and selects TLE sets from the Space-Track website provided by the North American Aerospace Defense Command (NORAD) [29], as shown in Table 1.

**Table 1:** The two-line element set of the LAGEOS-2 satellite.

1 22195U 92070B 24072.38712377 −.00000009 00000−0 00000−0 0 9990
2 22195 52.6585 69.1482 0137639 185.3165 325.9807 6.47293794741894

The TLE data are combined with the Simplified General Perturbations 4 (SGP4) model to perform orbital propagation and obtain the satellite’s orbital data. The three-dimensional position data are then compared with the high-precision Consolidated Prediction Format (CPF) ephemeris provided by the International Laser Ranging Service (ILRS) to calculate the position error (That is, SGP4 prediction error) [30]. The satellite orbital information provided by the CPF ephemeris has high accuracy, with typical errors within the meter range [31]. Therefore, the CPF ephemeris is used as the reference. The position error formula is shown in Equation (1).


$$\begin{array}{c} e=x_{CPF}\left(t\right)-x_{TLE}\left(t\right) \end{array}$$ (1)

$x_{CPF}\left(t\right)$ represents the position data from the CPF ephemeris, while $x_{TLE}\left(t\right)$ represents the position data predicted by the TLE combined with the SGP4 model.

**Fig 1:**
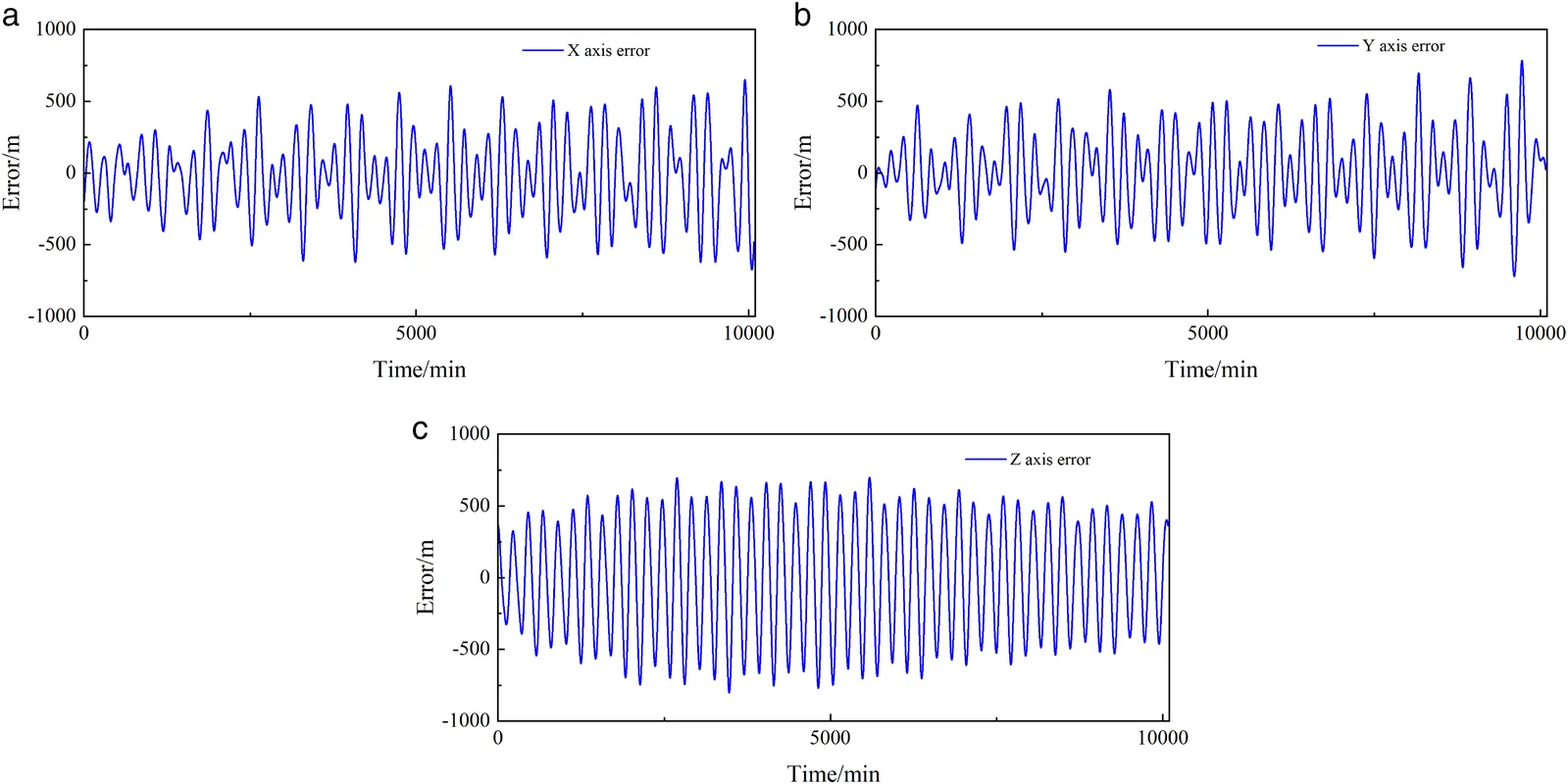
Positional error: (a) X-axis error, (b) Y-axis error, (c) Z-axis error.

**Fig 2:**
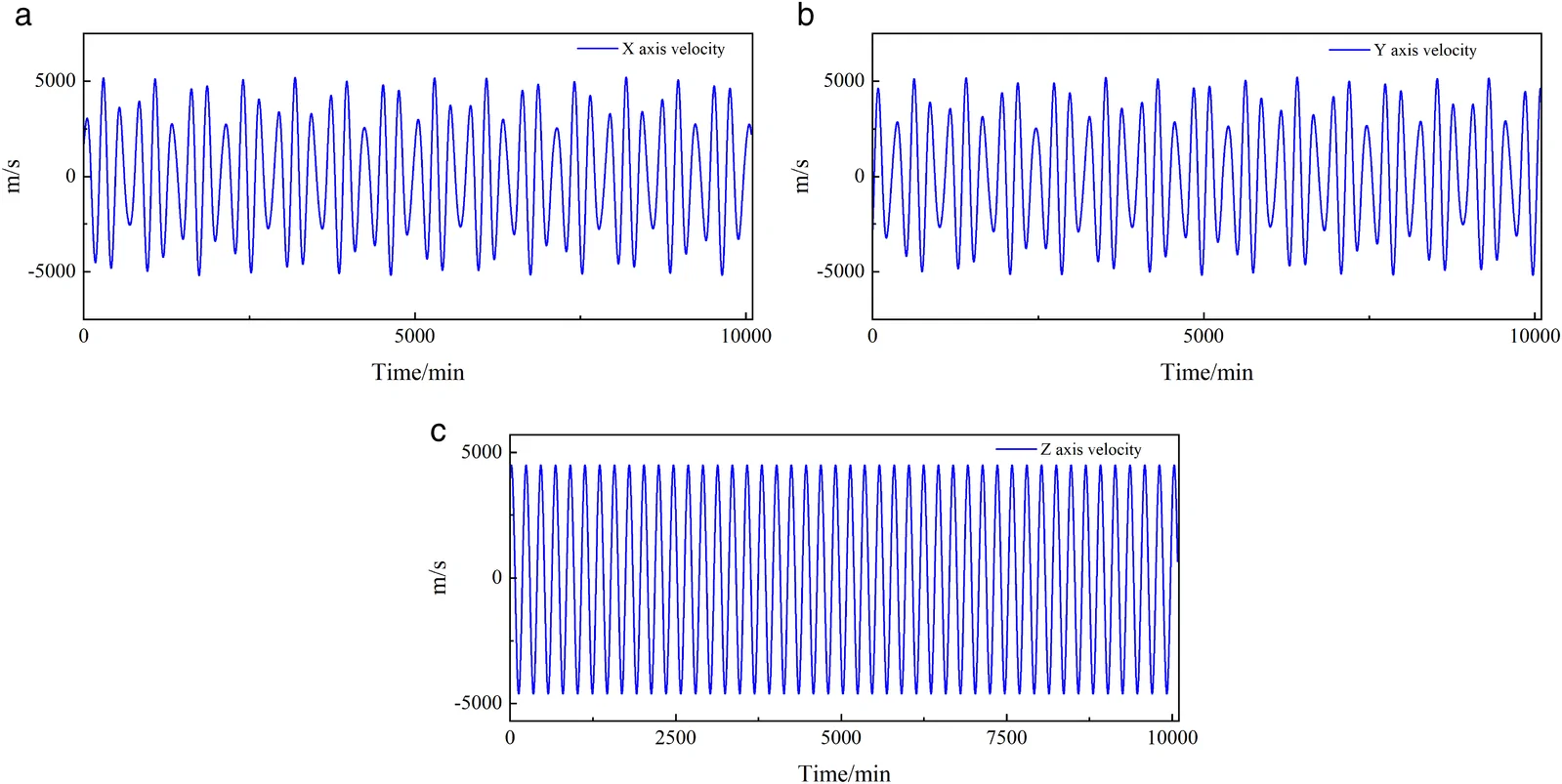
Satellite velocity: (a) X-axis velocity, (b) Y-axis velocity, (c) Z-axis velocity.

**Fig 3:**
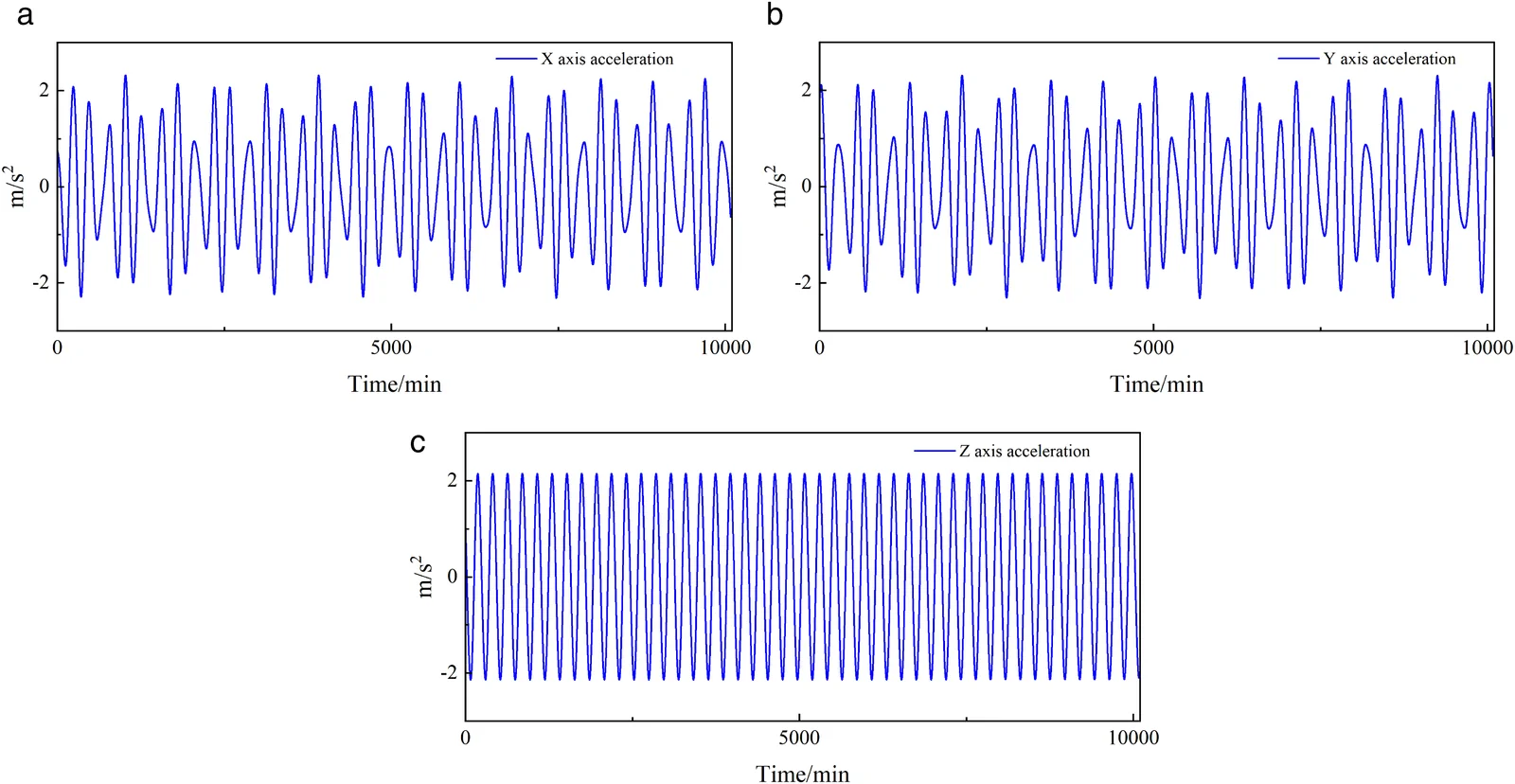
Satellite acceleration: (a) X-axis acceleration, (b) Y-axis acceleration, (c) Z-axis acceleration.

In satellite orbit prediction, the SGP4 model is an essential algorithm widely used for satellite orbit propagation. This model is typically employed together with the TLE data for orbit determination [32]. The TLE mainly provides orbital element sets collected for space objects. However, although the TLE data can provide a large volume of information, its precision is relatively low.In the SGP4 propagation model, the representation of the Earth’s gravitational field has certain limitations. It only includes the J2, J3, and J4 zonal harmonic coefficients to describe the perturbative effects of Earth’s gravity on satellite motion, while omitting the influence of the J22 tesseral harmonic component. When performing orbital propagation at the 10^2^ order of magnitude (normalized scale), this simplification may lead to prediction errors on the order of hundreds to even thousands of meters, thereby affecting the overall accuracy of the orbital forecast [33]. In this study, 7-day orbital propagation data starting from March 12, 2024, are used. The position error, velocity, and acceleration along the three orbital axes are shown in Figs 1–3, respectively.

In the X, Y, and Z axes, the position error exhibits significant fluctuations, with the error range approximately between ±1000 m. The velocity fluctuations are similar to the position error, with large variations, and the velocity range is between ±5200 m/s. Compared to position error and velocity, the acceleration shows smaller fluctuations, staying within the range of ±3 m/s^2^. Particularly in the Z axis, the acceleration changes are relatively smooth, and the overall fluctuations remain stable.

## 3 LSTM orbit prediction correction based on attention mechanism

### 3.1 Model design

**Fig 4:**
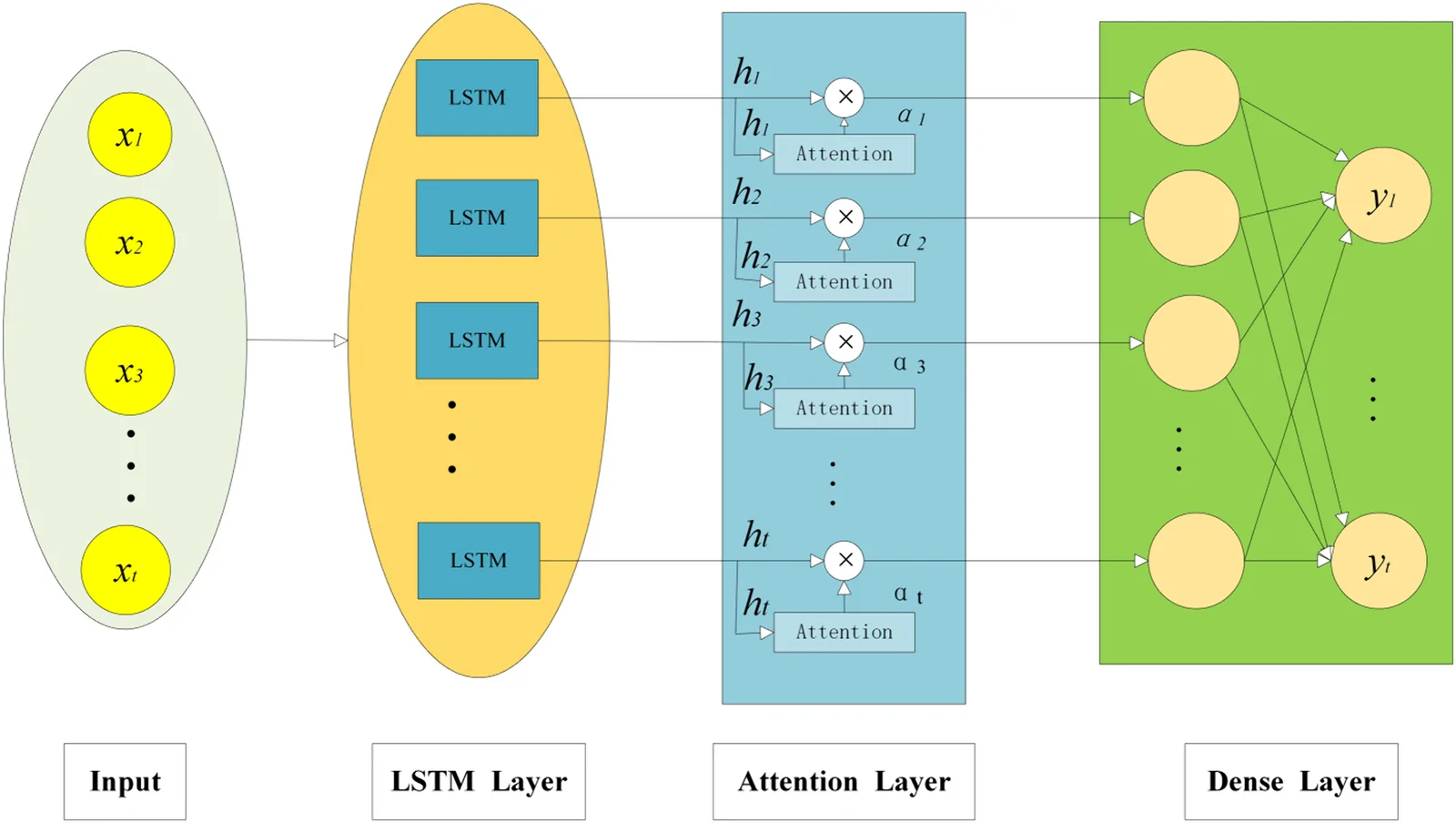
LSTM-attention mechanism architecture diagram.

The ATLSTM model is used for orbit prediction correction. LSTM achieves long-term memory through non-linear functions, capturing patterns in time series data, while the attention mechanism assigns weights across the entire sequence, highlighting key historical information to address the problem of long-term dependencies. The model consists of an input layer (position error, velocity, acceleration), an LSTM layer (extracting temporal patterns), an attention layer (focusing on important information), and a fully connected layer (generating the prediction results), all aimed at improving orbit prediction accuracy. The ATLSTM model is shown in Fig 4.

**Fig 5:**
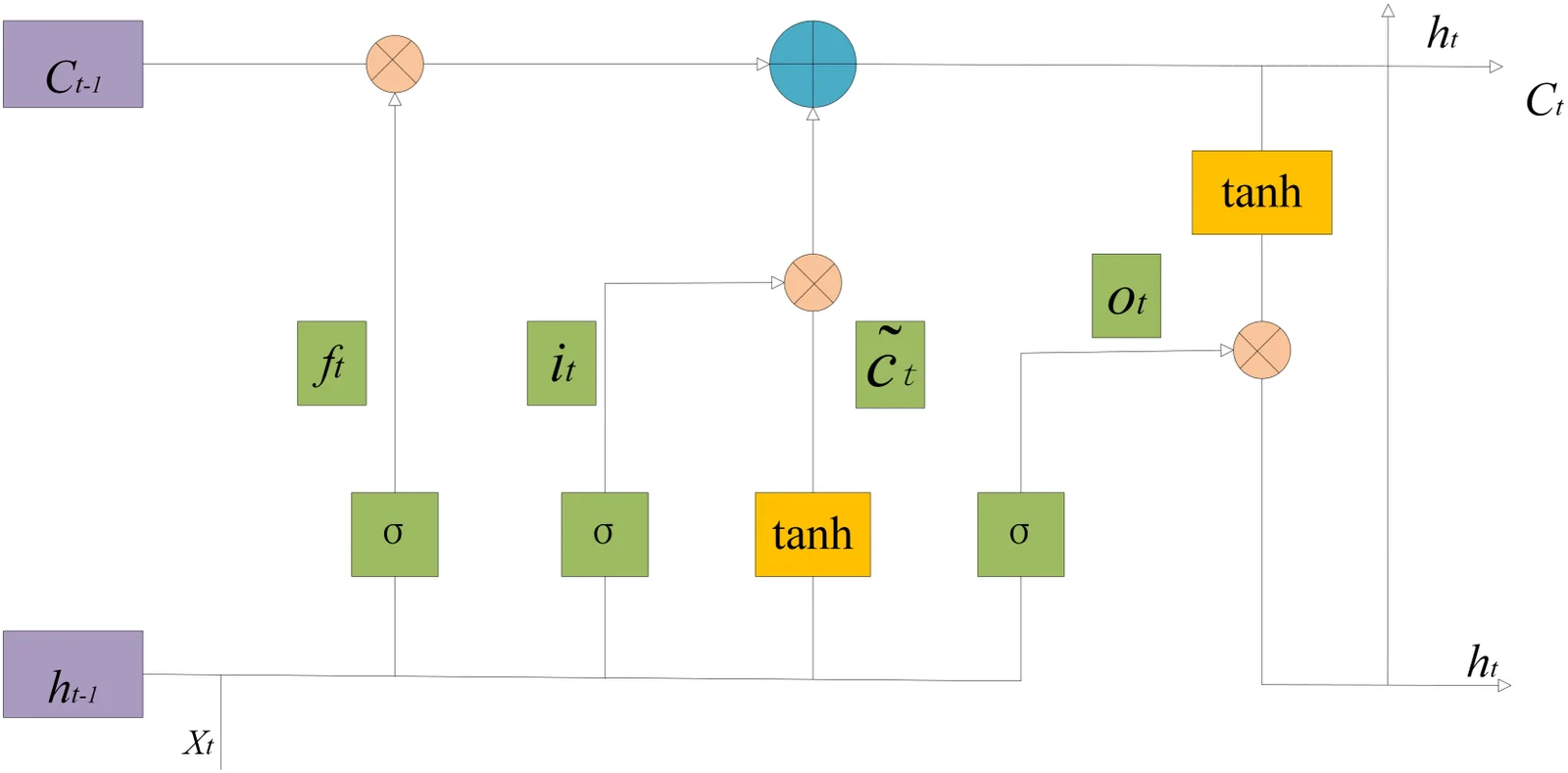
LSTM architecture diagram.

Long short-term memory is an improved version of the recurrent neural network (RNN), which can overcome the long-term dependency problem of RNN in long-sequence modeling. Its core structure consists of a memory cell, an input gate, a forget gate, and an output gate. Through the gating mechanism, LSTM realizes information selection and the learning of long-term dependencies. Its structure is shown in Fig 5.

The forget gate controls the degree to which the model retains or discards past information. It receives the current input $X_{t}$ and the output $h_{t-1}$ from the previous time step, and generates a vector $f_{t}$ with values between 0 and 1 through the sigmoid function σ, as shown in Equation (2). This vector represents the weight assigned to the content of the previous memory cell. The closer the value is to 1, the more information is retained.


$$\begin{array}{c} f_{t}=\sigma (w_{f}⬝x_{t}+U_{f}⬝h_{t-1}+b_{f}) \end{array}$$ (2)

The input gate is used to regulate the inflow of new information and determine the extent to which it updates the state of the memory cell. It consists of two sub-mechanisms. The first part uses the sigmoid function to determine the information that needs to be updated, as shown in Equation (3).


$$\begin{array}{c} i_{t}=\sigma (w_{i}⬝x_{t}+U_{i}⬝h_{t-1}+b_{i}) \end{array}$$ (3)

The other part uses the tanh function to generate the candidate information ${\tilde{C}}_{t}$, which is used to write into the memory cell, as shown in Equation (4). Then, the previous memory cell $C_{t-1}$ is combined with the candidate value to obtain the new memory state, as shown in Equation (5).


$$\begin{array}{c} {\tilde{C}}_{t}=tanh(W_{c}⬝x_{t}+U_{c}⬝h_{t-1}+b_{c}) \end{array}$$ (4)

$$\begin{array}{c} C_{t}=f_{t}⬝C_{t-1}+i_{t}⬝{\tilde{C}}_{t} \end{array}$$ (5)

The output gate uses the sigmoid function to generate an initial output vector $o_{t}$ between 0 and 1, as shown in Equation (6). Then, the memory cell state is mapped through the hyperbolic tangent function and multiplied element-wise by the activation value of the output gate to obtain the final output of the network, as shown in Equation (7).


$$\begin{array}{c} o_{t}=\sigma (W_{o}⬝h_{t-1}+W_{o}⬝x_{t}+b_{o}) \end{array}$$ (6)

$$\begin{array}{c} h_{t}=o_{t}⬝tanh\left(C_{t}\right) \end{array}$$ (7)

**Fig 6:**
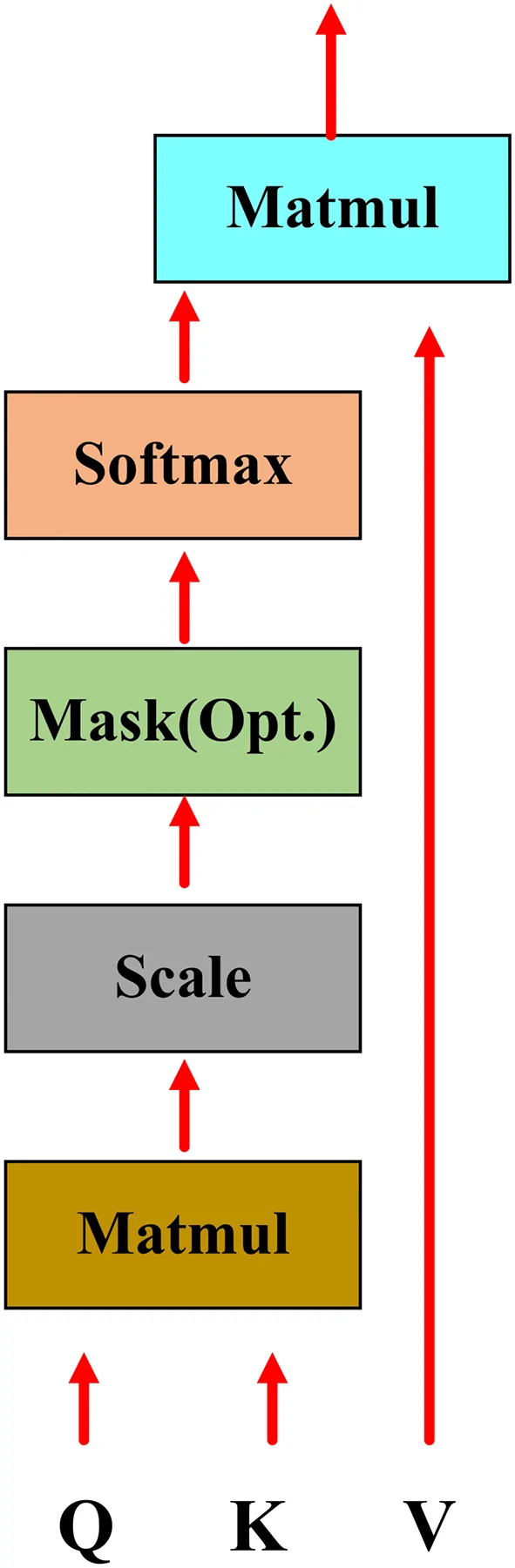
Attention mechanism architecture diagram.

The structure of the attention mechanism is shown in Fig 6. The attention mechanism first maps the hidden state sequence of the LSTM into the query matrix (Q), key matrix (K), and value matrix (V) through linear transformations. Let the hidden state sequence output by the LSTM layer be, $H=\left[h_{t-L+1},h_{t-L+2},\ldots ,h_{t}\right]\in R^{B\times L\times d_{h}}$, where B denotes the batch size, L denotes the length of the input time window, $d_{h}$ denotes the dimension of the LSTM hidden state, and $h_{t}$ denotes the LSTM hidden state at the t-th time step. For a single sample, H can be simplified as $H\in R^{B\times L\times d_{h}}$.

The Q, K, and V are obtained from the hidden state sequence (H) through linear transformations, respectively. The calculation processes are shown in Equations (8), (9), and (10).


$$\begin{array}{c} Q=HW_{Q} \end{array}$$ (8)

$$\begin{array}{c} K=HW_{K} \end{array}$$ (9)

$$\begin{array}{c} V=HW_{V} \end{array}$$ (10)

where $W_{Q}\in R^{d_{h}\times d_{k}}$, $W_{K}\in R^{d_{h}\times d_{k}}$, and $W_{V}\in R^{d_{h}\times d_{v}}$ are learnable weight matrices, which are used to map the input into appropriate dimensions. $d_{k}$ denotes the dimension of the query and key vectors, and $d_{v}$ denotes the dimension of the value vector. Therefore, $Q\in R^{B\times L\times d_{k}}$, $K\in R^{B\times L\times d_{k}}$, and $V\in R^{B\times L\times d_{V}}$.

Subsequently, the scaled dot-product attention mechanism is used to calculate the correlations among hidden states at different time steps. First, the dot product between the query matrix Q and the key matrix K is calculated and scaled by dividing by $\sqrt{d_{k}}$, yielding the attention score matrix S, as shown in Equation (11).


$$\begin{array}{c} S=\frac{QK^{T}}{\sqrt{d_{k}}} \end{array}$$ (11)

where $K^{T}$ denotes the transposition of the last two dimensions of K. The dimension of the attention score matrix S is $R^{B\times L\times L}$, which is used to represent the similarity relationships among different time steps.

Then, Softmax normalization is performed on S along the time-step dimension corresponding to the keys to obtain the attention weight matrix A, as shown in Equation (12).


$$\begin{array}{c} A=softmax\left(S\right)=softmax\left(\frac{QK^{T}}{\sqrt{d_{k}}}\right) \end{array}$$ (12)

where $A\in R^{B\times L\times L}$ denotes the attention weight matrix, and the elements in the matrix reflect the degree to which each time step attends to the hidden states of other historical time steps. It should be noted that this paper adopts the standard scaled dot-product attention mechanism with Softmax.

Finally, the attention weight matrix A is multiplied by the value matrix V to obtain the output representation O enhanced by the attention mechanism, as shown in Equation (13).


$$\begin{array}{c} O=AV=softmax\left(\frac{QK^{T}}{\sqrt{d_{k}}}\right)V \end{array}$$ (13)

where $O\in R^{B\times L\times d_{V}}$ is the output of the attention layer. For the prediction of the position error at time t+1, the attention output $O_{t}$ corresponding to the last time step is selected and mapped through the Dense layer to obtain the prediction result, as shown in Equation (14).


$$\begin{array}{c} {\hat{e}}_{t+1}=W_{d}O_{t}+b_{d} \end{array}$$ (14)

where ${\hat{e}}_{t+1}$ denotes the predicted position error at time t+1, $W_{d}$ is the weight matrix of the Dense layer, $O_{t}$ denotes the output representation of the attention layer at the t-th time step, and $b_{d}$ is the bias term.

Before model prediction, the input features need to be normalized. Normalization scales the position error, velocity, and acceleration data to a unified range, thereby eliminating the influence of differences in units and magnitudes and ensuring the stability and efficiency of model training. The normalization function used in this paper is shown in Equation (15).


$$\begin{array}{c} X_{m}=\frac{X_{origin}-X_{min}}{X_{max}-X_{min}} \end{array}$$ (15)

Here, $X_{origin}\text{,}X_{max}{\text{,X}}_{min}$ represents the original data, the maximum and minimum values in the training data; $X_{m}$ is the normalized value, with the processed data being restricted within the range (0, 1) to ensure that the network training process can converge quickly.

After the model prediction, to revert the normalized data back to its original scale, the predicted results need to be inverse normalized. The formula is shown in Formula (16).


$$\begin{array}{c} e_{ori}=e_{pre}(e_{max}-e_{min})+e_{min} \end{array}$$ (16)

$e_{pre}$ represents the predicted result from the fully connected layer, $e_{ori}$ refers to the predicted data after reverse normalization, $e_{max}$ represents the maximum value in the position error dataset, and $e_{min}$ represents the minimum value in the position error dataset.

To ensure the reproducibility of the model training process, the main structural parameters and training parameters of the MHALSTM model were set consistently, as shown in Table 2. Among them, the number of LSTM units and the number of attention heads are used to define the main network structure of the model. The time step represents the length of the historical sequence contained in each input window. The batch size, training epochs, learning rate, and L2 regularization coefficient are used to control the model training process. The number of input features corresponds to the three types of input variables selected in this paper, namely the SGP4 position error, velocity, and acceleration.

**Table 2:** Model parameter settings.

Model Parameter	Value
**Number of LSTM units**	64
**Number of attention heads**	1
**Time step**	150
**Batch size**	16
**Training epochs**	50
**L2 regularization coefficient**	0.01
**Learning rate**	0.001
**Optimizer**	Adam
**Number of input features**	3
**Number of output features**	1

The training process of a neural network largely determines the model’s performance. The principle is to measure the difference between the predicted results and the true labels using the loss function, and iteratively update the trainable weights through the backpropagation algorithm to gradually optimize the model, making its output closer to the true target or underlying distribution. Let the number of sample pairs in the dataset be n, and the definition of the model’s loss function is shown in Formula 17.


$$\begin{array}{c} loss=\frac{\text{1}}{n}{\sum_{i}^{n}\left(f\left(x_{i}\right)-y_{i}\right)}^{\text{2}}+\frac{\alpha }{2n}{\|w\|}_{\text{2}}^{\text{2}} \end{array}$$ (17)

Here, $x_{i}$ represents the input vector of the model, $f\left(x_{i}\right)$ refers to the output of the neural network model’s forward propagation, w represents the trainable parameters in the model, and α denotes the L2 regularization factor.

### 3.2 Input and output

The model constructed in this paper is a multivariate time series model, with inputs being the satellite’s position error, velocity, and acceleration from the previous 7 days, and the output being the predicted satellite position error. The specific expression is given by formula (18).


$$\begin{array}{c} X_{t}=[V\;A\;E]=\left(\begin{array}{ccc} {v^{\prime }}_{t-T+1} & {a^{\prime }}_{t-T+1} & {e^{\prime }}_{t-T+1}\\ \vdots & \vdots & \vdots \\ {v^{\prime }}_{t} & {a^{\prime }}_{t} & {e^{\prime }}_{t} \end{array}\right) \end{array}$$ (18)

$X_{t}$ is a matrix with T rows and 3 columns, where each row represents the data for one time step, including velocity, acceleration, and position error. There are T time steps of data in total.

### 3.3 Model training

To build a model for orbit prediction based on the historical orbital error data of the satellite’s three axes, a reasonable dataset needs to be designed. Since the dataset structure for all three axes is the same, the X-axis data in the ECEF (Earth-Centered, Earth-Fixed) coordinate system is selected, using continuous 8-day data from UTC (Universal Time Coordinated) 00:00:00 on March 12, 2024, to 00:00:00 on March 19, 2024, to design the training and test sets. The time step is set to 150, with each time step containing position error, velocity, and acceleration as features. The dataset is divided into a training set (the first 7 days, with 10,080 data points) and a test set (the last 1 day, with 1,440 data points). The data structure for the Y-axis and Z-axis is the same as that for the X-axis.

**Fig 7:**
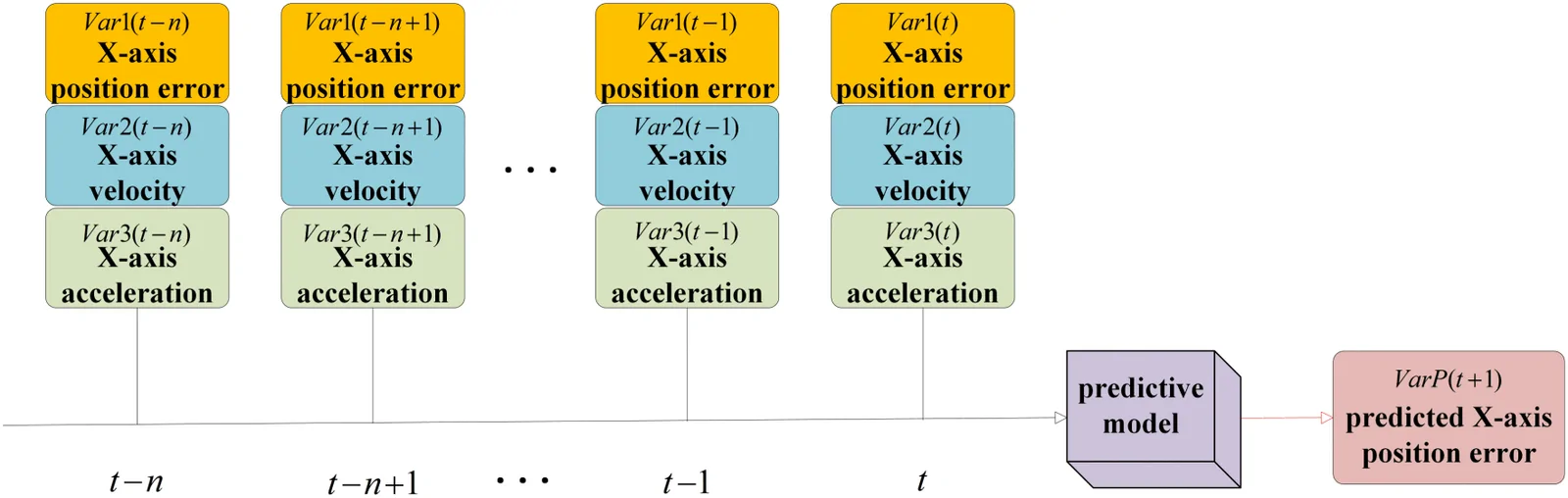
X-axis training data structure.

Each time step’s velocity, acceleration, and position error form a training data matrix $X_{t}$ based on the X-axis, which is then input into the prediction model to predict the position error for the next time step on the X-axis. Each training sample for the X-axis consists of the input features from the current time step and the corresponding label (Position error at time t+1), forming a set of input-label pairs $\left(X_{t},\left\{e_{t+1}\right\}\right)$. Since the training structure for the X-axis is the same as that for the other two axes, only the X-axis training structure in Fig 7 is shown here.

By using historical error data from a fixed time window as training data, the data is sequentially input into the ATLSTM model. The model performs forward propagation to predict the error for the next time step of the input sequence. During training, the model updates its weights by performing backpropagation based on the difference between the predicted position error and the actual position error. Each input sequence and its corresponding prediction result form a training sample. After completing one training step, the input sequence moves forward by one time step, and the process continues until the entire time-series data has been traversed.

### 3.4 Correction method

**Fig 8:**
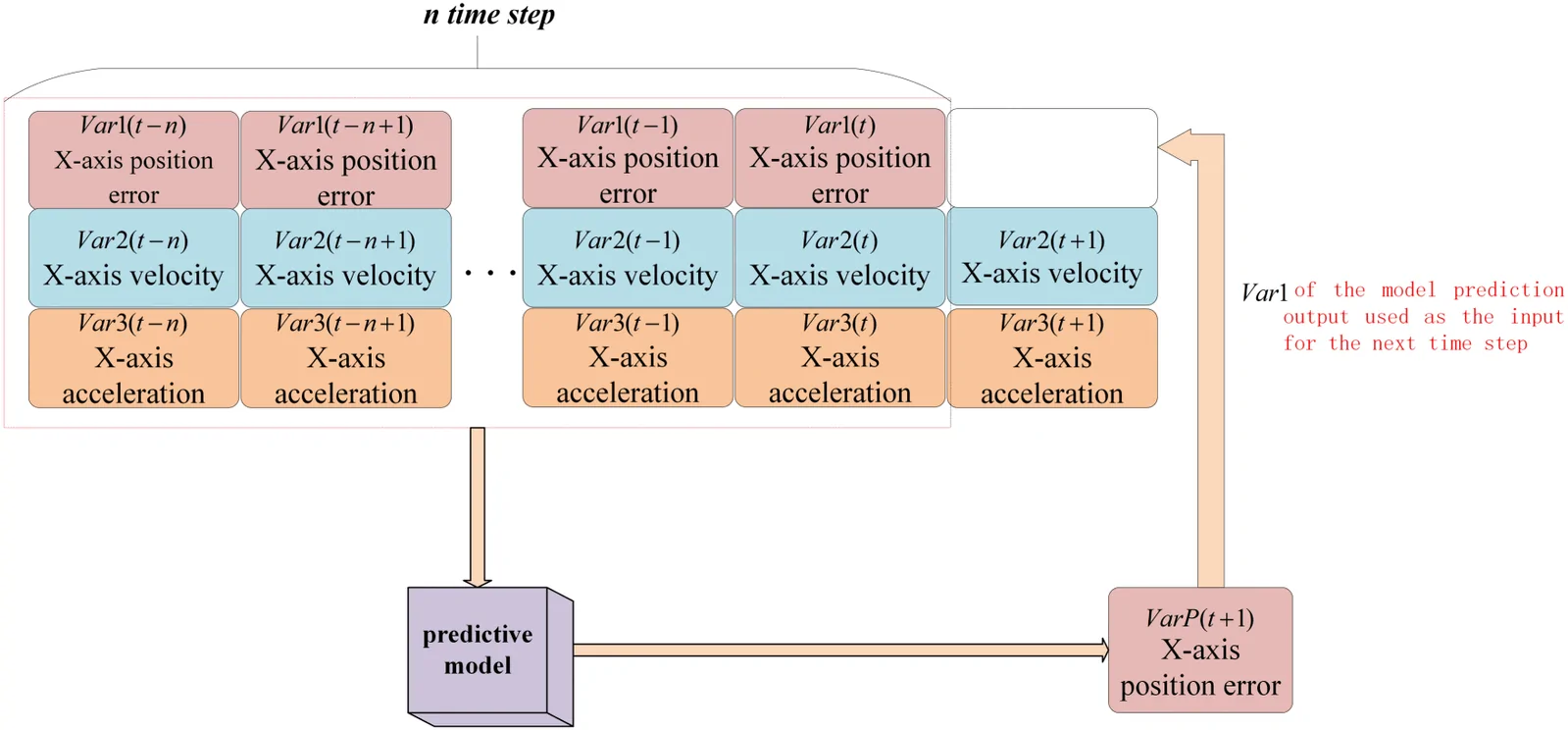
Prediction flowchart.


To validate the ATLSTM prediction accuracy, the model is used to predict the orbit position error for the next day based on the test set samples and compared with the actual errors. During the prediction process, the model performs only forward propagation without updating the parameters. Since the actual position errors after 00:00:00 on March 19, 2024, are unknown, the predicted values are used as substitutes. These values, along with velocity and acceleration (calculated using the SGP4 model), form new input data.The prediction process follows a recursive approach, starting from the last input sample of the training set. The model progressively predicts the position error for each time point and updates the data window. This method completes the error prediction over the entire time period. Using the predicted position errors ${\hat{e}}_{t+1}$, the original orbital data is corrected. The detailed prediction process is shown in Fig 7, and the corrected orbital prediction is shown in Fig 8. The correction formula for the position is provided in (19).

$$\begin{array}{c} x_{model}\left(t\right)=x_{TLE}\left(t\right)+{\hat{e}}_{t+1} \end{array}$$ (19)

### 3.5 Model evaluation

To evaluate the prediction accuracy of the model, this paper adopts the residual ratio P (Residual ratio, %) and the root mean square error (RMSE) as evaluation metrics. The calculation formulas are shown in Equations (20) and (21).


$$\begin{array}{c} p=\frac{\sum_{i=1}^{n}\left|e_{act}-e_{ATLSTM}\right|}{\sum_{i=1}^{n}\left|e_{act}\right|}\times 100\% \end{array}$$ (20)

where $e_{MHALSTM}$ represents the error predicted by the MHALSTM model, $e_{act}$ denotes the actual error, and n represents the number of prediction samples. A smaller P value indicates higher error prediction accuracy and better model performance.


$$\begin{array}{c} E_{RMSE}=\sqrt{\frac{\text{1}}{n}\sum_{i=1}^{n}(e_{act}-e_{ATLSTM}{)}^{\text{2}}} \end{array}$$ (21)

where n denotes the number of samples in the test set, $e_{act}$ represents the actual value, and $e_{MHALSTM}$ represents the predicted value. A smaller root mean square error indicates better model performance and higher accuracy.

In this paper, the residual ratio and RMSE are used to comprehensively evaluate model performance. The residual ratio is mainly used to measure the relative correction capability of the model for orbital errors, while RMSE reflects the absolute magnitude of the prediction errors and the overall prediction accuracy. By combining these two evaluation metrics, the performance of the ATLSTM model in orbital error prediction and correction tasks can be analyzed more comprehensively. Meanwhile, the residual ratio and RMSE are used to evaluate the model results, and the influence of different factors on the prediction performance of the ATLSTM model is further analyzed.

## 4 Experiments and results

### 4.1 Orbit prediction

**Fig 9:**
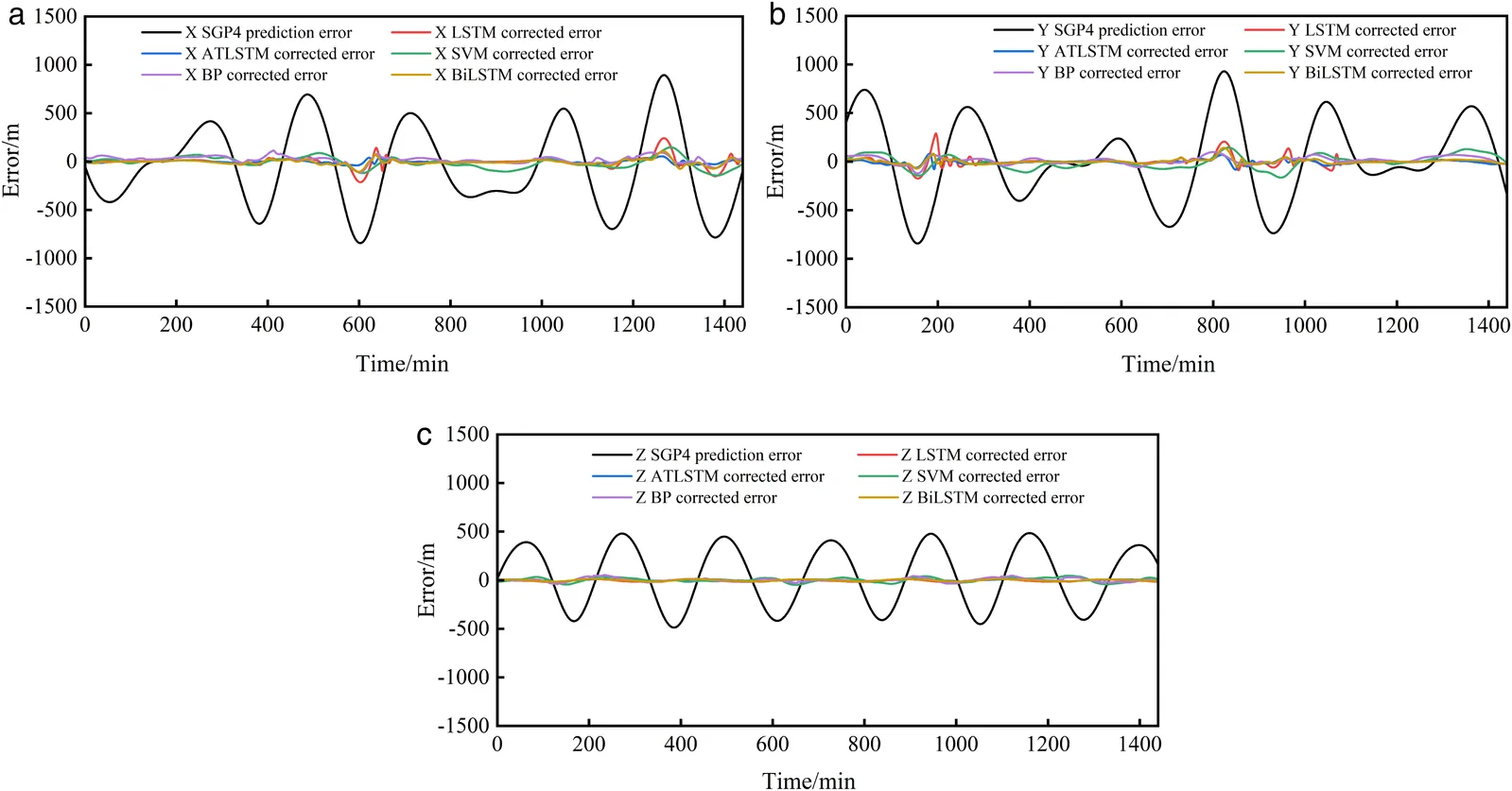
Model prediction results of orbital forecasting errors: (a) X-axis prediction, (b) Y-axis prediction, (c) Z-axis prediction.

Using three independently trained ATLSTM models, the SGP4 orbit prediction errors of the satellite were predicted and corrected, respectively. Fig 9 presents the orbital error correction results of different models on the test dataset, where Fig 9(a)–9(c) show the error variations in the X, Y, and Z directions of the ECEF coordinate system, respectively. In the figure, the black curve represents the position error predicted by SGP4, while the other colored curves represent the errors corrected by different models.

As can be seen from Fig 9, the original SGP4 orbit prediction errors exhibit obvious fluctuations in all three directions, with relatively large error amplitudes. Among them, the errors in the X- and Y-axis directions fluctuate more significantly, with peak values reaching several hundred meters and even approaching 1000 m in some local periods. The error variation in the Z-axis direction is relatively smooth, but still shows an obvious periodic deviation. After model correction, the errors in all directions clearly converge toward the vicinity of zero, indicating that the adopted error correction models can effectively suppress the systematic errors in SGP4 orbit prediction.

Further comparison of the correction results of different models shows that the error curves corrected by the ATLSTM model are generally smoother and have smaller fluctuation ranges. The model can better capture the variation trend of SGP4 orbit prediction errors and achieve effective compensation. Specifically, in the X- and Y-axis directions, the original SGP4 errors fluctuate significantly. After correction by the ATLSTM model, the errors are compressed to approximately ±50 m and ±80 m, respectively. In the Z-axis direction, the correction effect is more stable, and the error is basically compressed within ±15 m. The results indicate that the ATLSTM model has good error correction capability in all three directions of the ECEF coordinate system and can significantly improve the accuracy and stability of SGP4 orbit prediction.

**Fig 10:**
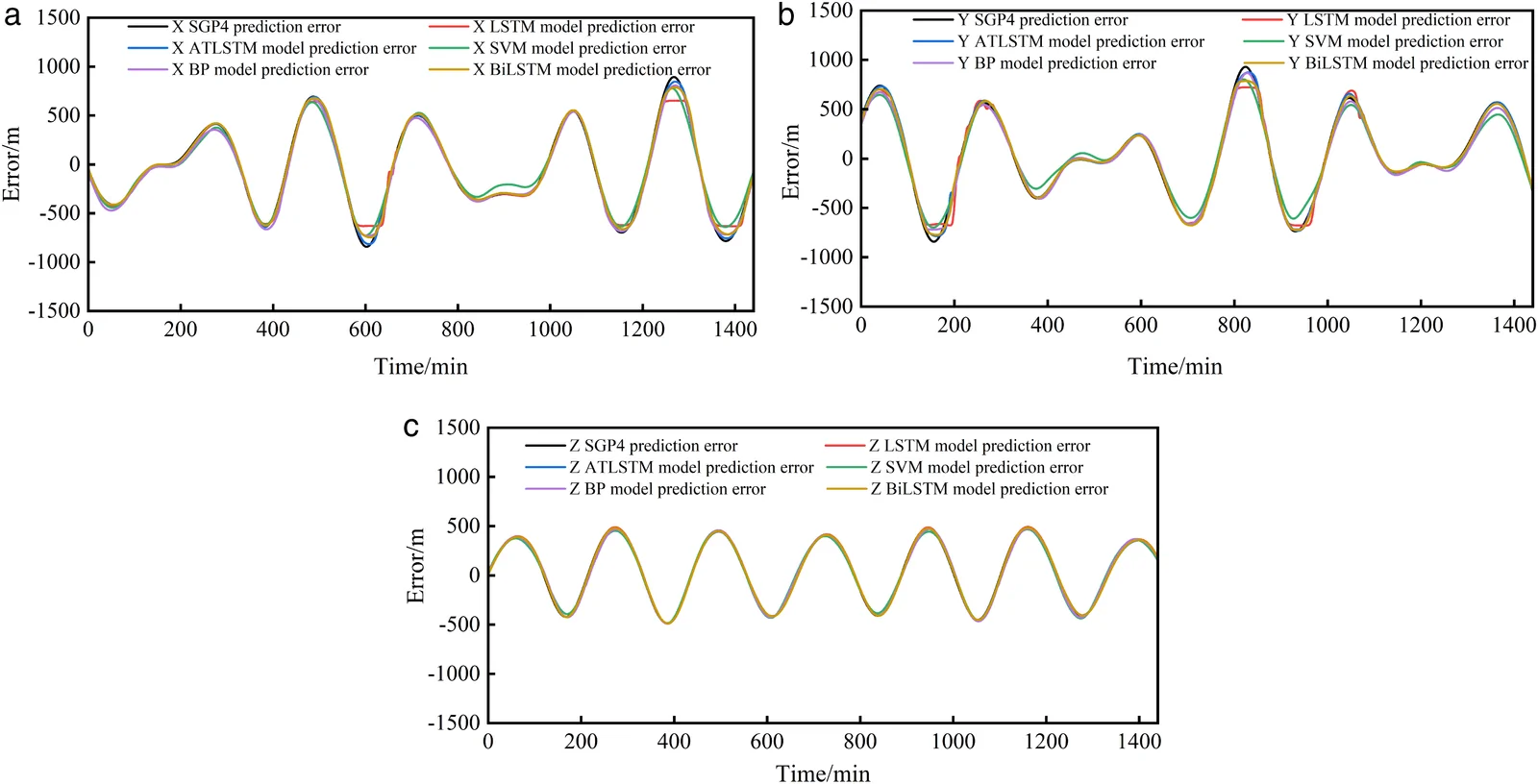
Prediction results comparison plot: (a) X-axis comparison, (b) Y-axis comparison, (c) Z-axis comparison.

Fig 10(a)–10(c) show the error prediction results of different models for the X, Y, and Z axes, respectively. Overall, the errors in all three directions show obvious fluctuations, and each model can follow the variation trend of the true errors to a certain extent, indicating that these models have certain time-series prediction capabilities. In the X- and Y-axis directions, the error fluctuations are relatively large, with obvious peak and valley variations, making the prediction relatively difficult. Compared with the other models, the prediction curve of ATLSTM agrees more closely with the true error curve. In particular, at positions where the errors change rapidly and reach local extrema, ATLSTM can describe the error variation trend more accurately. The LSTM, SVM, and BP models show certain deviations in some periods, while the BiLSTM model performs well overall, but its prediction accuracy is still slightly lower than that of ATLSTM. In the Z-axis direction, the error variation is relatively smooth, and the periodic pattern is more evident. The prediction results of different models are generally close. However, from the perspective of local details, ATLSTM shows better tracking performance at the peak and valley positions, and its prediction curve is closer to the true error. According to Table 3, the RMSE values of the ATLSTM model in the X, Y, and Z directions are 28.18 m, 21.84 m, and 7.59 m, respectively, all of which are lower than those of the other comparison models. The corresponding residual ratios values are 3.68%, 4.77%, and 2.37%, respectively, which are also at the lowest level. This indicates that the ATLSTM model has higher prediction accuracy and better stability in position error prediction.

**Table 3:** The comparison of RMSE and P values among different models.

Prediction Methods	X-axis		Y-axis		Z-axis	
	P	RMSE	P	RMSE	P	RMSE
**ATLSTM**	3.68	28.18	4.77	21.84	2.37	7.59
**LSTM**	8.17	54.20	7.81	49.71	3.01	9.78
**SVM**	12.71	58.92	16.53	69.19	6.91	23.01
**BP**	8.92	40.88	9.35	39.88	5.27	19.31
**BiLSTM**	5.39	28.59	5.09	26.05	2.67	8.52

In summary, the ATLSTM model demonstrates good performance in both position error prediction and position error correction. In terms of position error correction, the ATLSTM model can compensate for the original orbit prediction results based on the predicted errors, causing the corrected errors to converge significantly and improving the stability and accuracy of the orbit prediction results. In terms of position error prediction, the model can effectively capture the time-series variation characteristics of SGP4 orbit prediction errors, enabling the prediction results to maintain a high consistency with the true errors and demonstrating strong error modeling capability. Overall, the ATLSTM model can not only accurately predict the variation trend of position errors but also effectively improve the accuracy of SGP4 orbit prediction, showing good engineering application value.

### 4.2 The impact of hidden neuron units on the performance of the LSTM mModel

**Fig 11:**
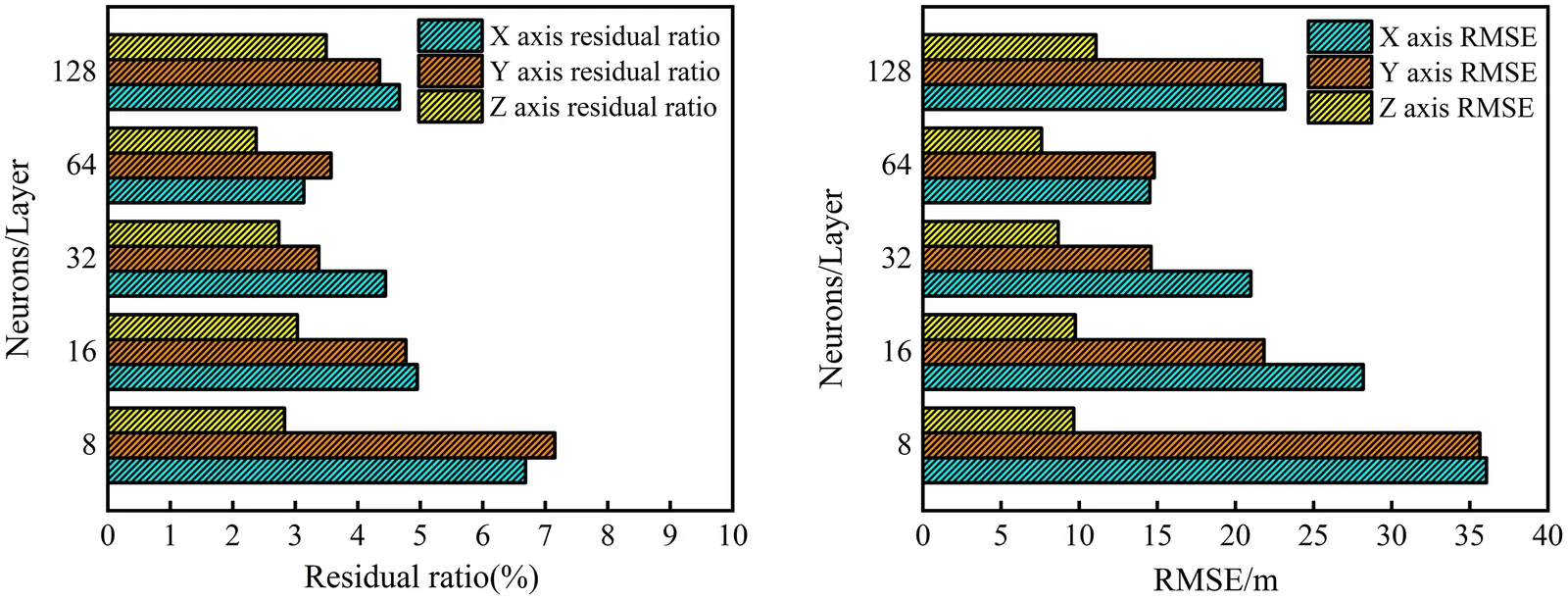
The RMSE values of ATLSTM under different neuron counts.

In deep learning, the number of neural units in the hidden layer is an important factor affecting the performance of neural networks. To optimize the model, it is necessary to investigate the influence of the number of hidden units on the training set and the test set. By adjusting the size of the hidden layer, the variation trend of model performance can be analyzed, so that the optimal number of hidden units can be determined. This process helps achieve a balance between increasing model complexity and maintaining good generalization capability, thereby improving the overall performance of the model. As shown in Fig 11, as the number of hidden neural units increases, the performance evaluation metrics of both methods decrease. Under the same number of hidden neural units, the performance differs among the three axes. For the X-axis, when the number of hidden neural units is 64, the model performs well, with the ATLSTM model achieving a residual ratio of 3.12% and an RMSE of 14.52 m. For the Y-axis, the best performance is obtained when the number of neural units is 32, with a residual ratio of 3.38% and an RMSE of 14.60 m. For the Z-axis, the best performance is achieved when the number of neural units is 64, with a residual ratio of 2.37% and an RMSE of 7.59 m. The introduction of the attention mechanism significantly enhances the stability and prediction accuracy of the model, especially in the Z-axis direction. The experimental results show that, in neural network design, reasonably selecting the number of hidden units and effectively applying the attention mechanism are crucial for improving model performance.

### 4.3 The impact of different training durations on model prediction results

**Fig 12:**
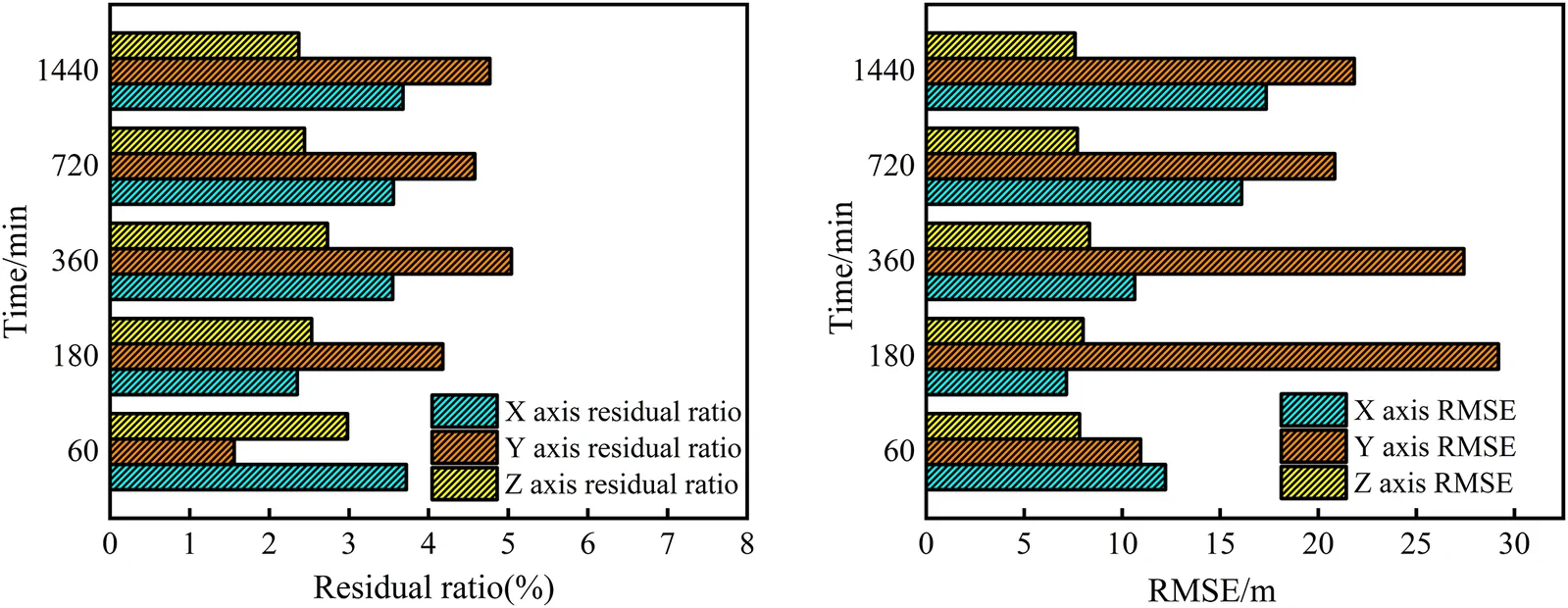
Model prediction performance at different time intervals (min).

**Fig 13:**
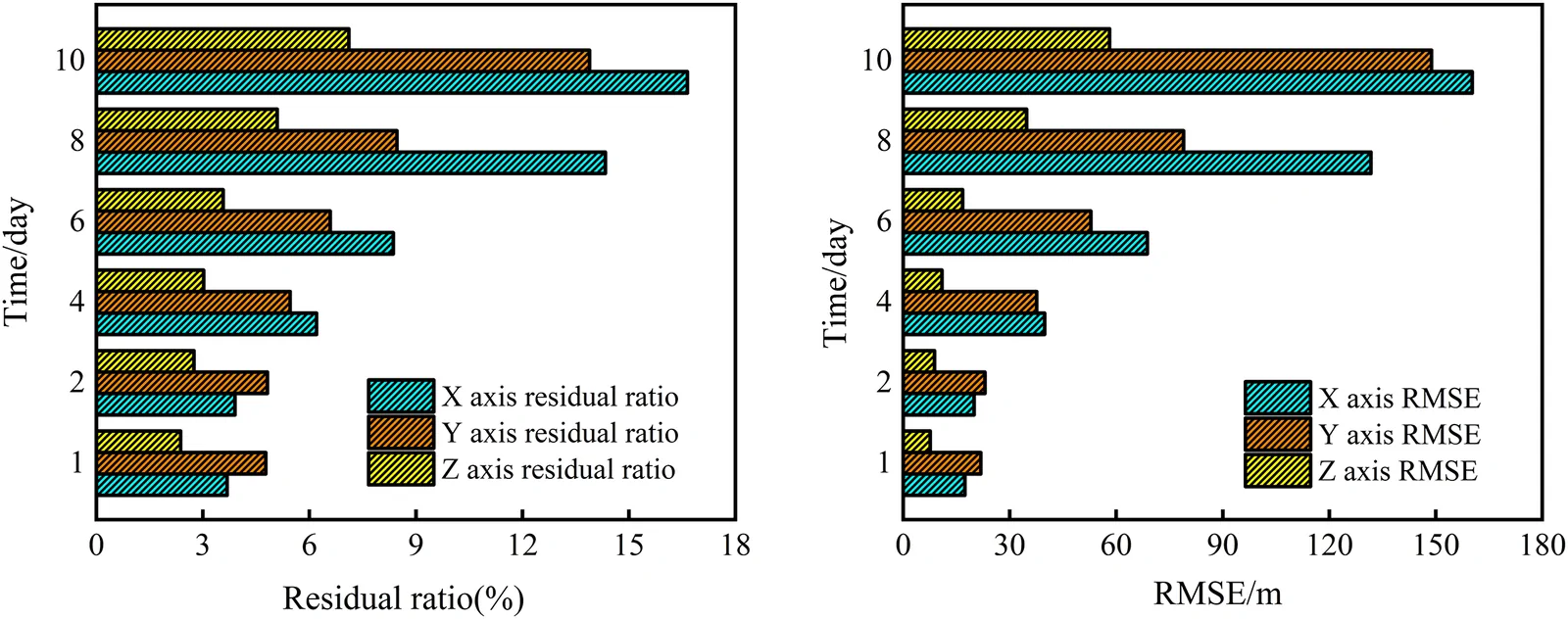
Model prediction performance for different prediction days.

To analyze the influence of different time scales on the orbital error prediction performance of the ATLSTM model, experiments are conducted from two aspects: different time intervals within one day and different prediction days. The model performance is evaluated using the residual ratio and RMSE. The results are shown in Tables 4 and 5, and Fig 12 and 13.

**Table 4:** Model prediction performance at different time intervals (min).

Evaluation Metrics	Residual ratio(%)			RMSE/m		
Time/min	X-axis	Y-axis	Z-axis	X-axis	Y-axis	Z-axis
**60**	3.72	1.56	2.98	12.20	10.93	7.82
**180**	2.35	4.18	2.53	7.15	29.19	8.00
**360**	3.55	5.04	2.73	10.63	27.43	8.33
**720**	3.56	4.58	2.44	16.09	20.84	7.71
**1440**	3.68	4.77	2.37	17.35	21.84	7.59

**Table 5:** Model performance metrics for different prediction days.

Evaluation Metrics	Residual ratio(%)			RMSE/m		
Time/day	X-axis	Y-axis	Z-axis	X-axis	Y-axis	Z-axis
**1**	3.68	4.77	2.37	17.35	21.84	7.59
**2**	3.91	4.82	2.75	19.94	23.02	8.80
**4**	6.20	5.46	3.02	39.83	37.60	10.90
**6**	8.36	6.58	3.57	68.76	52.82	16.71
**8**	14.33	8.46	5.09	131.74	78.93	34.73
**10**	16.64	13.89	7.11	160.23	148.81	58.14

First, five time intervals, namely 60 min, 180 min, 360 min, 720 min, and 1440 min, are selected to analyze the influence of different time scales within one day on the prediction performance of the ATLSTM model. As shown in Table 4 and Fig 12, at different minute scales, the model maintains low residual ratios in the X, Y, and Z directions, indicating that the ATLSTM model has good correction capability for orbital errors under different time intervals. Specifically, the residual ratio in the X direction ranges from 2.35% to 3.72%, with the best performance achieved at 180 min; the residual ratio in the Y direction varies relatively significantly, ranging from 1.56% to 5.04%; and the residual ratio in the Z direction is relatively stable, remaining between 2.37% and 2.98%. In terms of RMSE, the minimum value in the X direction is 7.15 m at 180 min; the minimum value in the Y direction is 10.93 m at 60 min; and the RMSE in the Z direction changes only slightly, remaining between 7.59 m and 8.33 m. Overall, under different time scales within one day, the ATLSTM model shows relatively stable prediction performance, among which the Z direction is least affected by changes in the time interval, while the Y direction exhibits relatively larger fluctuations.Furthermore, six prediction lengths, namely 1 day, 2 days, 4 days, 6 days, 8 days, and 10 days, are set to analyze the influence of different prediction days on model performance. As shown in Table 5 and Fig 13, as the number of prediction days increases, the residual ratios and RMSE values of the model in all three directions generally show an upward trend, indicating that error accumulation occurs during long-term prediction. For 1-day and 2-day predictions, the model errors are relatively small, demonstrating good short-term prediction capability. When the prediction days increase to 4 days and 6 days, the RMSE values in the X and Y directions increase more noticeably. When the prediction length reaches 8 days and 10 days, the model prediction errors further increase. For the 10-day prediction, the RMSE values in the X, Y, and Z directions reach 160.23 m, 148.81 m, and 58.14 m, respectively, and the corresponding residual ratios are 16.64%, 13.89%, and 7.11%, respectively.

In summary, the ATLSTM model shows relatively stable overall prediction performance at different minute scales within one day and can effectively correct orbital prediction errors. However, as the number of prediction days increases, model errors gradually accumulate and the prediction accuracy decreases. Therefore, the ATLSTM model is more suitable for short-term orbital error prediction and correction, while its ability to model long-term error variation characteristics still needs to be further improved for predictions over longer time scales.

### 4.4 Generalization capability of the model

To further verify the applicability of the proposed ATLSTM model to different orbital targets, two additional satellites with different orbital altitudes are selected for orbital error prediction and correction experiments based on the original experiments. These satellites are HY-2B, with an orbital altitude of approximately 971 km, and LARES-2, with an orbital altitude of approximately 5899 km. Experiments on satellites at different orbital altitudes can further examine the adaptability and generalization performance of the model for data with different orbital characteristics.

**Fig 14:**
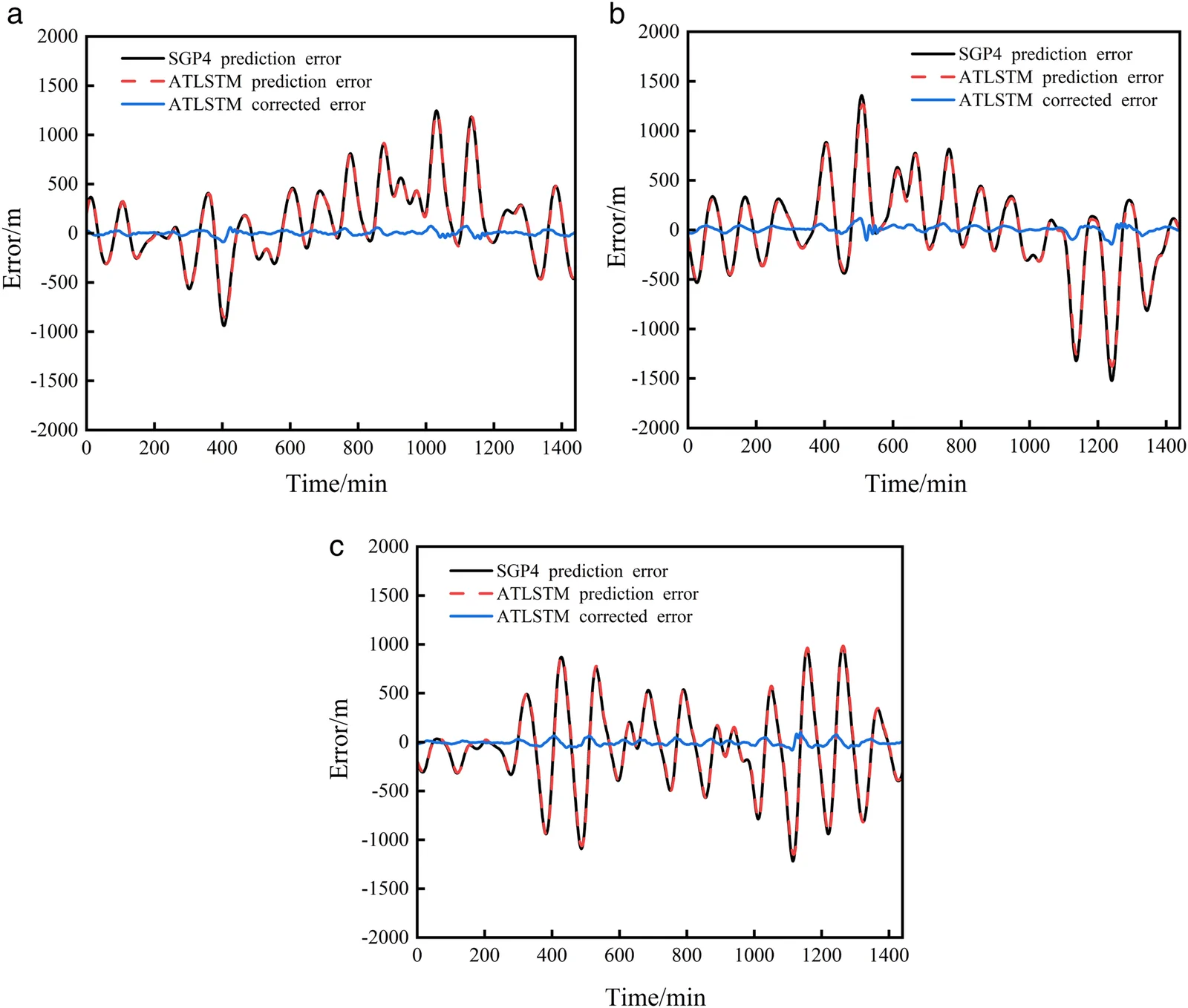
Orbit prediction results of the HY-2B satellite: (a) X-axis prediction, (b) Y-axis prediction, (c) Z-axis prediction.

**Fig 15:**
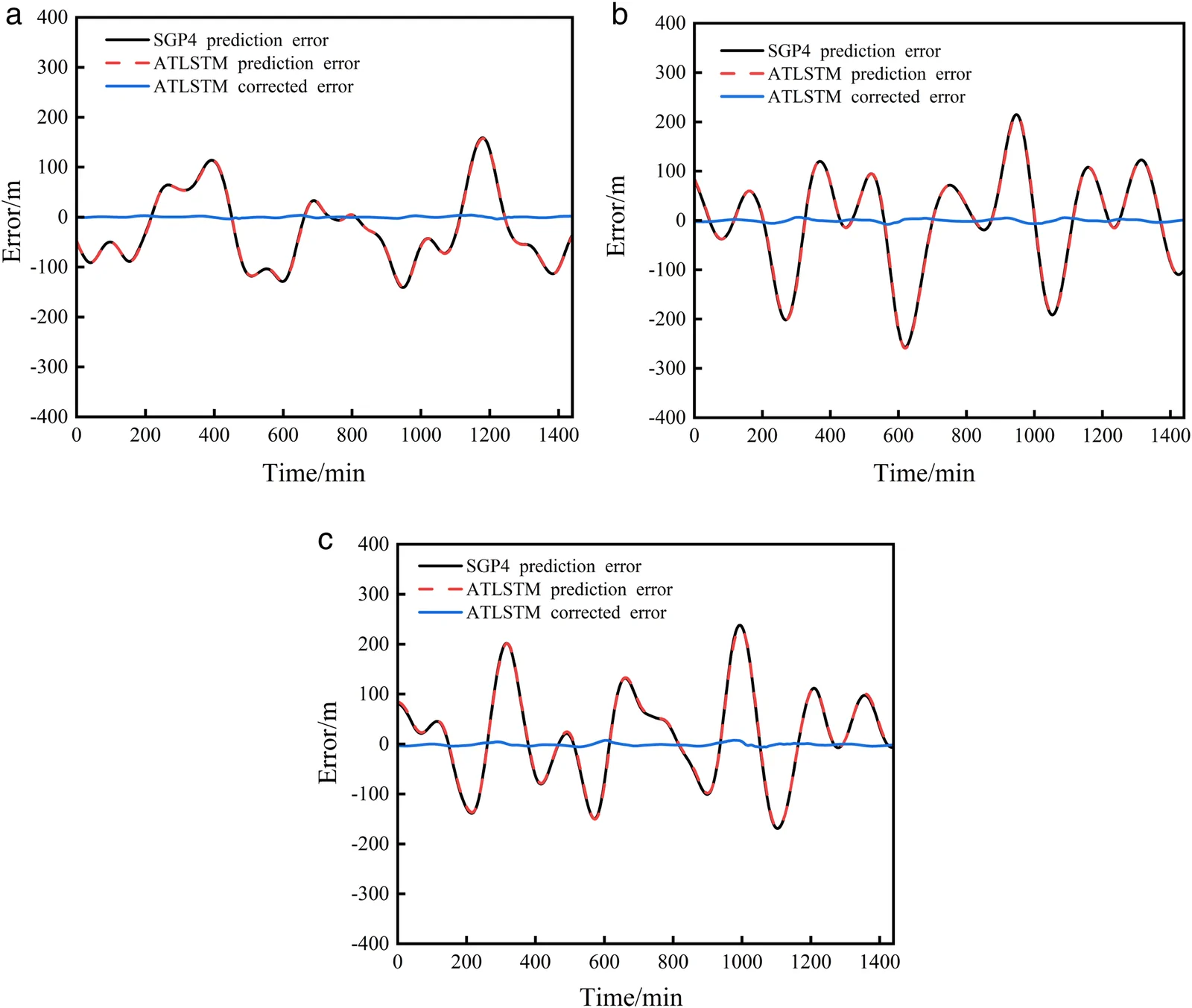
Orbit prediction results of the LARES-2 satellite: (a) X-axis prediction, (b) Y-axis prediction, (c) Z-axis prediction.

Fig 14 presents the orbital error prediction results of the HY-2B satellite in the X, Y, and Z coordinate axes. As shown in the figure, the prediction errors of the SGP4 model exhibit obvious fluctuations in all three directions, while the ATLSTM model can effectively learn the variation trend of the errors. After model correction, the orbital errors in the three directions are significantly reduced, and the corrected error curves are generally closer to the vicinity of zero. Specifically, the position errors of the HY-2B satellite in the X, Y, and Z directions are compressed to 89.92 m, 144.53 m, and 100.32 m, respectively. As shown in Table 6, the residual ratios of the HY-2B satellite in the X, Y, and Z directions are 5.95%, 8.15%, and 6.81%, respectively, and the corresponding RMSE values are 23.99 m, 36.48 m, and 28.58 m. These results indicate that the ATLSTM model can effectively correct the orbital prediction errors of the HY-2B satellite. Fig 15 presents the orbital error prediction results of the LARES-2 satellite in the X, Y, and Z coordinate axes. Compared with the HY-2B satellite, the original orbital prediction errors of the LARES-2 satellite are generally smaller. However, after correction by the ATLSTM model, the residual errors are further reduced, and the corrected error curves also remain near zero. Specifically, the position errors of the LARES-2 satellite in the X, Y, and Z directions are compressed to 4.00 m, 7.84 m, and 7.38 m, respectively. The evaluation metrics in Table 6 show that the residual ratios of the LARES-2 satellite in the X, Y, and Z directions are 1.79%, 3.06%, and 3.43%, respectively, with corresponding RMSE values of 1.60 m, 3.03 m, and 3.11 m. These results demonstrate that the ATLSTM model also has good prediction and correction capability for satellite targets with higher orbital altitudes and smaller error amplitudes.

**Table 6:** Evaluation metrics of satellites.

Satellite	HY-2B			LARES-2		
Coordinate axis	X-axis	Y-axis	Z-axis	X-axis	Y-axis	Z-axis
**Residual ratio(%)**	5.95	8.15	6.81	1.79	3.06	3.43
**RMSE/m**	23.99	36.48	28.58	1.60	3.03	3.11

Based on the experimental results of the HY-2B and LARES-2 satellites, it can be seen that the proposed ATLSTM model is not only applicable to the LAGEOS-2 satellite used in the original experiment, but can also be applied to other satellite targets at different orbital altitudes. For both the HY-2B satellite with an orbital altitude of approximately 971 km and the LARES-2 satellite with an orbital altitude of approximately 5899 km, the model can effectively learn the variation patterns of SGP4 orbit prediction errors and significantly reduce the corrected residual errors. These results indicate that the ATLSTM model has good generalization capability and can maintain relatively stable orbital error prediction and correction performance under different orbital altitudes and different error amplitude conditions.

## 5 Conclusion

To address the strong dependence of space-object orbit prediction on physical models and initial conditions, as well as the difficulty of completely eliminating prediction errors, this study proposes a satellite orbit prediction error correction method that combines an attention mechanism with a long short-term memory network. Taking the LAGEOS satellite as the research object, an attention-based long short-term memory model is constructed using historical orbital position errors, velocity, and acceleration as input features to predict and correct the orbit prediction errors generated by SGP4.The experimental results show that the ATLSTM model can effectively improve the accuracy of one-day-ahead orbital position error prediction. The maximum position errors along the X-, Y-, and Z-axes are reduced to 54.20 m, 83.18 m, and 14.89 m, respectively, and the Residual ratios in all three directions are reduced to below 5%. Compared with the LSTM, SVM, BP, and BiLSTM models, the ATLSTM model demonstrates higher prediction accuracy and better stability.Further analysis indicates that both the number of hidden-layer neurons and the prediction horizon affect model performance. Selecting an appropriate number of neurons helps improve the predictive capability of the model. However, as the number of prediction days increases, error accumulation becomes progressively more pronounced, indicating that the proposed model is more suitable for short-term orbital error prediction and correction. Validation results obtained for the HY-2B and LARES-2 satellites show that the ATLSTM model possesses a certain degree of adaptability and generalization capability for different space objects.It should be noted that this study still has certain limitations. The number of satellite targets included in the experiments is limited, error accumulation remains an issue in long-term prediction, and the applicability of the proposed method to real-world operational space situational awareness scenarios requires further validation. Future work will expand the range of satellite samples, incorporate additional features, and improve methods for suppressing long-term error accumulation, thereby enhancing the stability and applicability of the model in complex orbit prediction scenarios.

In conclusion, the orbit prediction method integrating deep learning and an attention mechanism demonstrates considerable potential for improving the accuracy of space-object orbit prediction and provides theoretical and technical support for enhancing space situational awareness capabilities and the reliability of orbital collision avoidance.
